# From COVID-19-Associated Acute Kidney Injury to Long-Term Kidney Dysfunction: Recovery Phenotypes, Risk Stratification, and Post-COVID Renal Surveillance

**DOI:** 10.3390/pathogens15101022

**Published:** 2026-09-29

**Authors:** Faisal Madkhali, Yahia A. Mjery, Mostafa Mohrag, Mohammed Abdulrasak

**Affiliations:** 1 Department of Medical Laboratory Technology, College of Nursing and Health Sciences, Jazan University, Jazan 45142, Saudi Arabia; fmadkhali@jazanu.edu.sa (F.M.); ymjery2@jazanu.edu.sa (Y.A.M.); 2 Department of Internal Medicine, College of Medicine, Jazan University, Jazan 45142, Saudi Arabia; mmohrag@jazanu.edu.sa; 3 Department of Clinical Sciences, Lund University, 205 02 Malmö, Sweden; 4 Department of Gastroenterology and Nutrition, Skåne University Hospital, 205 02 Malmö, Sweden

**Keywords:** acute kidney injury, chronic kidney disease, COVID-19, post-acute COVID-19 syndrome, renal recovery

## Abstract

Background: COVID-19-associated acute kidney injury (AKI) is common in severe disease, but its long-term kidney implications are heterogeneous and incompletely defined. Objective: To synthesize recovery phenotypes and longitudinal outcomes, reconcile conflicting evidence on whether COVID-associated AKI differs from other-cause AKI, and set out implications for surveillance and management. Methods: The evidence base was assembled through a targeted PubMed/MEDLINE search for COVID-19 and kidney dysfunction (January 2020 to August 2026), hand-searching of reference lists, and direct retrieval of pivotal longitudinal cohorts and clinical practice guidelines. Longitudinal cohorts and comparative studies were prioritized. Findings: AKI severity, duration, kidney replacement therapy, lower baseline estimated glomerular filtration rate (eGFR), and incomplete early recovery consistently identify patients at greatest risk. Longitudinal studies disagree on whether COVID-AKI confers more risk than AKI from other illnesses: early cohorts reported steeper eGFR loss, whereas a later 9624-patient cohort found lower adjusted major adverse kidney events. This divergence reflects differences in kidney reference point, outcome construction, ascertainment, and handling of competing death rather than genuine contradiction. Population studies show long-term kidney failure is concentrated after hospitalization and severe disease. Candidate biomarkers remain investigational. Conclusions: Post-COVID kidney risk is severity- and phenotype-dependent rather than universal. Follow-up is most defensible after hospitalized AKI, incomplete recovery, advanced chronic kidney disease (CKD), dialysis, or transplantation and applies existing post-AKI guidance rather than a COVID-specific schedule. Survivors who meet Kidney Disease: Improving Global Outcomes (KDIGO) criteria for CKD at three months, namely a GFR below 60 mL/min/1.73 m^2^ or a persistent marker of kidney damage, become eligible for guideline-directed therapy, though no trial has enrolled on recent AKI. Prospective studies should test whether structured follow-up improves outcomes.

## 1. Introduction

The acute kidney manifestations of COVID-19 are well described. The unresolved problem is which survivors fail to recover, whether their trajectory differs from AKI caused by other illnesses, and how post-discharge care should be targeted. AKI is heterogeneous rather than a single exposure: severity, duration, baseline reserve, critical illness, and the definition and timing of recovery all alter prognosis. Consensus terminology separates AKI within 7 days from acute kidney disease (AKD) at 7–90 days and CKD beyond 90 days, but COVID-19 studies apply these inconsistently [1].

Several contemporary reviews already cover ‘COVID-19 and the kidney’ broadly: a 2024 AKI update, 2025 scoping and narrative reviews of renal long COVID, a 2025 Nature Reviews Nephrology synthesis, and a 2026 review of infection and vaccination, together addressing incidence, pathology, long-term outcomes, and vaccine-associated events [2,3,4,5,6]. A further contribution therefore requires a narrower thesis. This review focuses on the AKI-to-AKD/CKD continuum, reconciles discordant longitudinal studies, separates validated predictors from exploratory biomarkers, and proposes a risk-stratified follow-up framework. Two contributions distinguish it. First, rather than reporting the conflicting comparative studies side by side, it identifies the design features accounting for their divergence, making the disagreement interpretable rather than merely noted. Second, it carries the analysis through to management, asking what should be done for survivors whose dysfunction persists and where guideline-directed therapy already applies, a question that existing syntheses do not address.

## 2. Scope and Approach

This is a narrative review. It is not a systematic review, and the distinction is material. A systematic review applies prespecified eligibility criteria through duplicate screening, grades each included study with a validated risk-of-bias instrument, and pools estimates where appropriate; a narrative synthesis selects and weighs studies by explicit but author-applied criteria and reports the reasoning rather than a pooled estimate. This review is the latter, and the sections that follow are written so that the reasoning can be audited. The evidence base was assembled in three ways. First, a targeted PubMed/MEDLINE search combining COVID-19 with the phrase “kidney dysfunction” in the title or abstract, restricted to publications from 2020 onward, was run in August 2026; the exact query, its yield, and the records selected from it are reported in Supplementary Materials Figure S1. At revision, a second, structured PubMed/MEDLINE query combining COVID-19 and acute kidney injury with recovery and long-term outcome terms (Humans; Adult; English; 2020–2026) was run as a reproducibility check; it returned 324 records and independently retrieved 8 of the 11 principal longitudinal and comparative cohorts on which the argument rests; abstract screening of the 75 retrieved records whose titles indicated longitudinal or kidney function outcomes identified thirteen eligible studies not previously included, which have been incorporated, and its string, yield, overlap with the reference list, and screening counts are reported in Supplementary Materials. Second, the primary longitudinal and comparative cohorts on which the argument rests were identified by hand-searching the reference lists of the recent reviews cited in Section 1 and of the primary studies retrieved, supplemented by the authors’ knowledge of the literature; this step, rather than the database search, supplied most of the included primary studies. Third, clinical practice guidelines and studies raised during peer review were retrieved directly. The database search was deliberately narrow and served to identify recent reports not already known to the authors; it did not, and was not designed to, enumerate the field, and this is declared as a limitation in Section 12.

Records were eligible if they reported original data in adults on kidney function trajectories, recovery, kidney failure, proteinuria, or major adverse kidney events after COVID-19. Mechanistic pathology series and biomarker cohorts were eligible where they informed persistent dysfunction or risk stratification. Studies were excluded if restricted to pediatric populations, if they reported only in-hospital outcomes without post-discharge follow-up, if they were case reports or series of fewer than ten patients, if they were non-human or in vitro studies, or if they were published in a language other than English. Pediatric exclusion reflects the distinct epidemiology, AKI definitions, and baseline creatinine assumptions of that population; the language restriction is a limitation. Records were assessed against these criteria and the prioritization rules in Supplementary Materials; the database yield and the composition of the final reference set are reported there. Screening, selection, extraction, and interpretation were performed by one author (Y.A.M.) without duplicate assessment, and no validated risk-of-bias instrument was applied. Bibliographic details and every numerical estimate cited were verified against PubMed records and, where accessible, primary reports.

Among eligible studies, those given most weight in the synthesis were selected on prespecified design grounds rather than on the direction of their findings. Comparative designs with a contemporaneous non-COVID comparator were prioritized over single-arm cohorts, because the central question of the review is whether COVID-associated AKI differs from AKI of other cause; multicenter or population-level studies over single-center series; longer follow-up over shorter; designs anchored to a pre-illness kidney baseline over those using admission or discharge values; and analyses that handled competing death explicitly over those that did not. Where studies produced contradictory results, they were not weighted by vote-counting or by sample size alone. Each contradiction was first interrogated for differences in the four design features that Section 6 identifies as decisive, namely kidney reference point, survivor conditioning, mode of ascertainment, and handling of competing death, and was treated as a genuine contradiction only if those differences did not account for it. These four features are recorded for every major cohort in Section 5 so that the weighting can be checked against the studies themselves. The conclusions of the review therefore rest on design reasoning that a reader can follow rather than on a pooled estimate, and they carry the limitations of author judgment set out in Section 12.

An AI-based large language model (Claude, Anthropic; model version Claude Fable 5.1) assisted with language editing, reference formatting, figure drafting, and abstract-level screening of the records retrieved by the supplementary structured search at revision; all eligibility and inclusion decisions were made by the authors, and the model was not used to extract data or draw conclusions (see Declarations).

## 3. COVID-19-Associated Acute Kidney Injury as the Starting Point

Early cohorts established a steep severity gradient. Among 5216 hospitalized US veterans, 32% developed AKI, with markedly higher mortality and ventilation in that group; a large New York cohort likewise showed rising adjusted mortality with increasing AKI severity and dialysis requirement [7,8]. Compared with influenza, COVID-19 brought more frequent and severe AKI, although survivor recovery was more similar than crude outcomes suggested [9]. These cohorts predate widespread vaccination and are not timeless incidence estimates.

What matters at discharge is not whether AKI occurred but its phenotype, developed in Section 5. In the STOP-COVID cohort, lower baseline eGFR and oliguria at kidney replacement therapy (KRT) initiation were strongly associated with non-recovery in critically ill patients [10]. Death is a competing event; restricting analyses to survivors can make recovery look favorable while excluding the most severely injured.

## 4. Mechanisms Linking Acute Injury to Persistent Dysfunction

COVID-19-associated AKI is multifactorial, and the mechanisms proposed for it differ greatly both in how well they are established and in whether they are specific to SARS-CoV-2 at all. Because mechanistic plausibility is easily mistaken for evidence of causation, this section organizes the candidate mechanisms into three tiers: those shared with critical-illness AKI of any cause, for which COVID-19 is one trigger among many; those for which SARS-CoV-2 may plausibly amplify a shared pathway without evidence that it does so uniquely; and those for which a COVID-specific causal trigger has been established. The tiers are summarized in Table 1. The distinction matters because the central argument of this review, that long-term outcomes after COVID-associated AKI resemble those after other severe AKI, depends on the first tier dominating; the evidence indicates that it does.

### 4.1. Mechanisms Shared with Critical-Illness AKI

Acute tubular injury is the dominant lesion, arising from hypoxemia, hemodynamic instability, venous congestion, systemic inflammation, endothelial dysfunction, and nephrotoxic exposures. Proximal-tubule dysfunction with low-molecular-weight proteinuria was common in a detailed hospitalized cohort [11], and autopsy series described tubular injury, casts, and vascular congestion [12]. None of these lesions is unique to COVID-19; each is characteristic of sepsis and of critical-illness AKI generally. Three lines of evidence place them firmly in this tier. First, in an observational cohort of 223 consecutive patients, severe AKI occurred exclusively in intensive care, 97% of those affected had respiratory failure, and mechanical ventilation, vasopressor therapy, and rising inflammatory markers were the independent time-varying predictors, so that severe AKI tracked overall illness severity rather than any kidney-specific process [13]. Second, single-nucleus transcriptomics of kidneys from patients dying with severe AKI identified the same four injury-associated epithelial cell states, characterized by oxidative stress, hypoxia, interferon response, and epithelial-to-mesenchymal transition, whether or not the patient had COVID-19 [14]. Third, biomarker signatures of severe AKI overlap between COVID-19 and non-COVID respiratory illness [15], and recovery among survivors was similar to that after influenza-associated AKI once crude hospital outcomes were set aside [9]. The pathways of persistence after severe AKI of any cause, namely incomplete tubular repair, capillary rarefaction, interstitial inflammation, fibroblast activation, recurrent injury, and progression of pre-existing diabetic, hypertensive, or vascular disease, belong to this tier as well.

### 4.2. Mechanisms Plausibly Amplified by SARS-CoV-2

A second group of pathways is shared with other critical illness but may be quantitatively intensified by SARS-CoV-2 on the basis of features that appear more prominent in COVID-19 than in comparable illness without being unique to it. Coagulopathy and microthrombosis are the clearest candidates: microthrombi were described in early autopsy series [12], and a thrombotic phenotype was a prominent feature of severe COVID-19, although its magnitude relative to that of bacterial sepsis has not been formally quantified. Endothelial injury and complement activation, including complement deposition on the tubular brush border, were prominent in early pathology [16]. Sustained immune and endothelial activation after the acute phase has been proposed to intensify the persistence pathways described above, although evidence for a distinct human fibrotic program remains incomplete [17]. Direct viral infection of tubular epithelium also belongs in this tier rather than the third, and its history illustrates why the tiering is necessary.

Direct kidney infection remains contested, and the trajectory of that debate is itself instructive. Early in the pandemic, comprehensive syntheses assembled a coherent virus-centered hypothesis: angiotensin-converting enzyme 2 (ACE2) is abundantly expressed on proximal tubular cells and podocytes, early autopsy series reported viral protein in tubular epithelium with complement deposition on the brush border, and electron microscopy identified intracellular structures interpreted as coronavirus-like particles, so that direct viral cytopathy appeared a plausible primary driver of kidney injury [16]. That framework was reasonable on the evidence then available, and it shaped the first wave of clinical interpretation. Subsequent biopsy series using in situ hybridization found no definitive viral signal and argued against direct infection as the dominant mechanism, and the specificity of the electron microscopy findings was questioned [18]. A later meta-analysis found frequent acute tubular injury but variable, method-dependent viral detection, with heterogeneity and postmortem sampling limiting causal inference [19]. The field has therefore moved from a virus-centered model toward one in which renal viral material may be detectable in some severe cases, but systemic and host-response pathways explain much of the observed AKI. Stating that evolution explicitly matters for the argument of this review: if the dominant mechanisms are those of critical illness rather than of the virus itself, the long-term trajectory after COVID-associated AKI should resemble that after other severe AKI, which is what the comparative data increasingly suggest (Section 6).

For each mechanism in this tier, the defensible statement is the same: SARS-CoV-2 may amplify a pathway that other severe illnesses also engage. None has been shown to operate only in COVID-19, and none has been shown to alter long-term kidney outcomes relative to AKI of other cause.

### 4.3. Mechanisms with an Established COVID-Specific Trigger

The third tier is small. COVID-19-associated collapsing glomerulopathy is the one entity for which SARS-CoV-2 infection has been established as the specific precipitant. Case series linked collapsing lesions and nephrotic-range proteinuria to high-risk apolipoprotein L1 (APOL1) genotypes, characteristically without demonstrable virus in kidney tissue, supporting a two-hit model in which infection-driven interferon signaling injures podocytes in genetically susceptible hosts [20,21]. Two qualifications apply even here. The lesion is not unique to SARS-CoV-2: the same collapsing phenotype is recognized after HIV infection and after therapeutic interferon in APOL1 high-risk individuals, so what is COVID-specific is the trigger, not the mechanism, and it is rare and confined to a genetically defined subgroup, so it cannot account for the long-term trajectory of the majority of survivors. Race should not proxy for genotype; social exposures, access to care, comorbidity, and ancestry-related genetic risk must be separated analytically.

**Table 1:** Proposed mechanisms of COVID-19-associated AKI assigned to three evidence tiers: shared with critical-illness AKI of any cause; plausibly amplified by SARS-CoV-2 without evidence of a unique role; or with an established COVID-specific trigger.

Mechanism	Tier	Basis for Assignment	Key Evidence
Hypoxemia, hemodynamic instability, venous congestion	Shared	Universal in critical illness; severe AKI tracked ventilation and vasopressor use	[13]
Systemic inflammation, endothelial dysfunction	Shared	Characteristic of sepsis; rising inflammatory markers predicted severe AKI	[13]
Nephrotoxic exposure	Shared	Drug and contrast exposure common to intensive care	—
Acute tubular injury, proximal tubulopathy	Shared	Dominant lesion; identical epithelial injury states with and without COVID-19	[11,12,14]
Incomplete repair, capillary rarefaction, fibrosis	Shared	Persistence pathways after severe AKI of any cause	[17]
Coagulopathy, microthrombosis	Plausibly amplified	Microthrombi in autopsy series; thrombotic phenotype prominent in severe COVID-19	[12]
Complement activation, endothelialitis	Plausibly amplified	Complement deposition on tubular brush border in early pathology	[16]
Sustained post-acute immune activation	Plausibly amplified	Proposed; no distinct human fibrotic program demonstrated	[17]
Direct tubular infection	Plausibly amplified (contested)	Variable, method-dependent viral detection; not the dominant mechanism	[16,18,19]
Collapsing glomerulopathy in APOL1 high-risk genotype	COVID-specific trigger	Two-hit model; interferon-driven podocyte injury in susceptible hosts; virus typically absent from tissue	[20,21]

**Figure 1:**
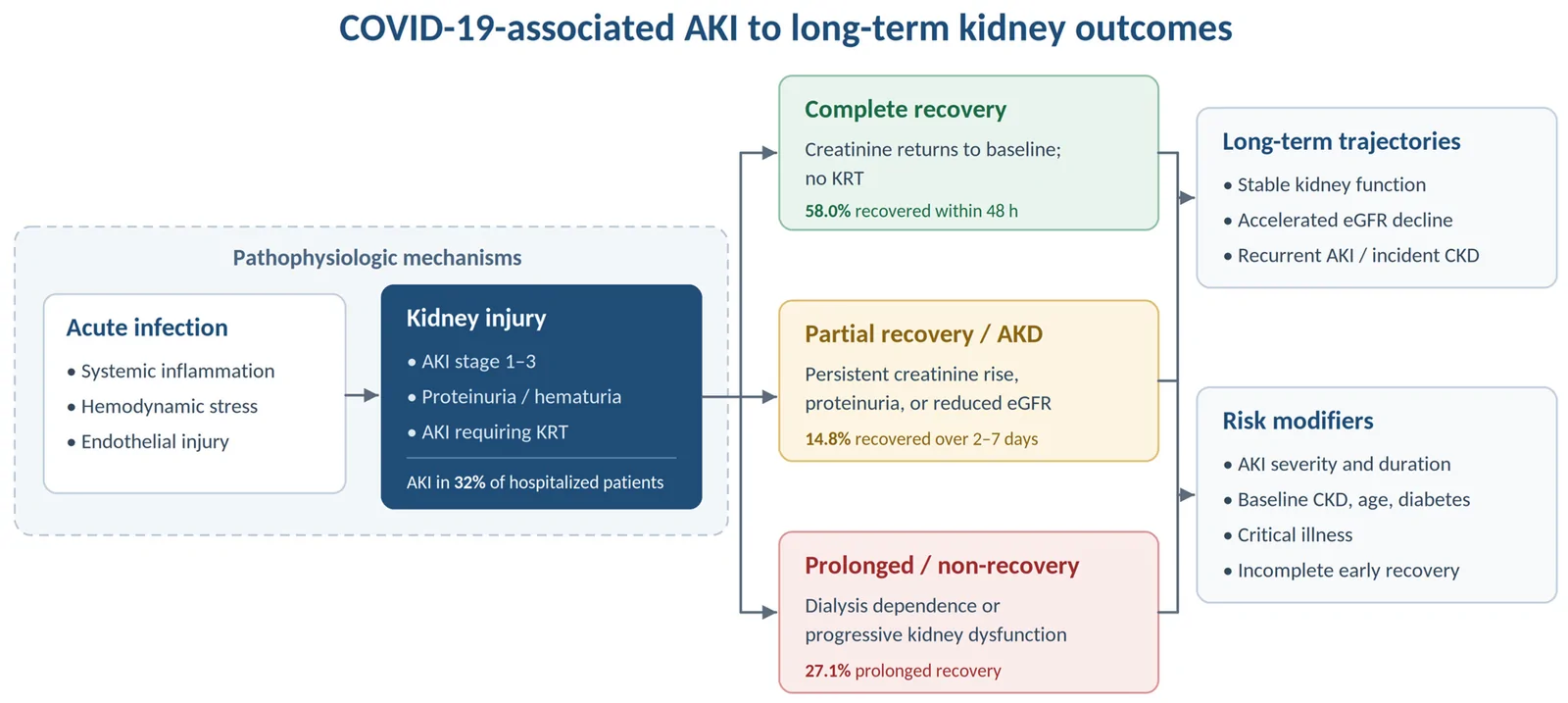
Conceptual pathway from COVID-19-associated AKI to recovery, AKD, CKD, or kidney failure. Percentages denote time to recovery rather than final outcome state and are drawn from Lu et al. [22] (*n* = 3296 hospitalized COVID-19 survivors with AKI: 58.0% recovered within 48 h, 14.8% over 2–7 days, and 27.1% had prolonged recovery). AKI incidence is from Bowe et al. [7] (*n* = 5216 hospitalized US veterans). Arrows indicate plausible clinical pathways, not established causality. Abbreviations: AKD, acute kidney disease; AKI, acute kidney injury; CKD, chronic kidney disease; eGFR, estimated glomerular filtration rate; KRT, kidney replacement therapy.

Taken together, the tiering yields a clear conclusion. The mechanisms that account for most COVID-associated AKI are those of critical illness; the mechanisms that may be amplified by SARS-CoV-2 have not been shown to change outcomes; and the one mechanism with an established COVID-specific trigger is rare and genetically restricted. Mechanistic plausibility should therefore complement, not substitute for, comparative longitudinal evidence (Figure 1).

## 5. Recovery Phenotypes and the Transition to Chronic Kidney Disease

Recovery is not binary. Useful phenotypes include rapid reversal within 48 h, delayed recovery over 2–7 days, persistent AKD through 90 days, dialysis independence with residual eGFR loss, and non-recovery with ongoing KRT. A small early study of KRT-requiring COVID-AKI survivors reported favorable dialysis independence, but its survivor-only denominator limits generalization [23]; the dialysis-requiring phenotype is considered separately in Section Dialysis-Requiring AKI and the Transition to Maintenance Dialysis. A five-hospital comparison later found steeper adjusted post-discharge eGFR decline after COVID-AKI [24].

Timing of recovery is prognostically informative. Among 3296 survivors with hospital AKI, 58.0% recovered within 48 h, 14.8% recovered within 2–7 days, and 27.1% had prolonged recovery, which was associated with more 90-day major adverse kidney events (MAKE), recurrent AKI, and new CKD [22]. Among 12,891 hospitalized patients internationally, stage 3 AKI was strongly associated with persistent creatinine elevation at 180 and 365 days; in the subset without baseline CKD, age, male sex, and hypertension also marked higher risk [25]. By contrast, moderate/severe AKI with and without COVID-19 showed similar 12-month incident or progressive CKD in the small survivor subgroup, suggesting critical illness and AKI severity explain much of the risk [26]. In 1734 Wuhan survivors followed for a median of 342 days, AKI during the acute phase was associated with an 8.3% greater percentage decline in eGFR and 4.6-fold higher odds of reduced kidney function at follow-up, with a graded effect from stage 1 to stage 3 [27].

These studies support a trajectory-based rather than disease-label approach. Peak creatinine alone is insufficient: duration of injury, discharge creatinine relative to baseline, residual albuminuria, and KRT dependence are actionable. Definitions should acknowledge uncertainty in baseline creatinine, dilution and muscle-loss effects, and competing death. Table 2 summarizes the cohorts and syntheses informing these trajectories, applying to each the four design features that Section 6 shows to govern their interpretation.

### Dialysis-Requiring AKI and the Transition to Maintenance Dialysis

The dialysis-requiring phenotype warrants separate consideration because its outcome distribution differs qualitatively from that of non-dialysis AKI and because it is the phenotype most easily misread. Dialysis-requiring COVID-associated AKI follows a bimodal course: high early mortality followed, among survivors, by a high rate of dialysis independence.

The in-hospital picture is consistent across cohorts. In the STOP-COVID study of 4221 adults admitted to intensive care at 68 US hospitals, 876 (21%) developed AKI requiring KRT; of these, 588 (67%) died, 95 (11%) were discharged without kidney recovery, and 193 (22%) recovered by discharge, so that one-third of survivors left hospital still dialysis-dependent. Lower baseline eGFR and oliguria at KRT initiation were each independently associated with non-recovery, with odds ratios rising to 8.69 for baseline eGFR of 15 mL/min/1.73 m^2^ or below and 4.02 for urine output below 50 mL/day [10]. In the 13-hospital New York cohort, 30.6% of survivors of dialysis-requiring AKI remained on dialysis at discharge, and pre-existing CKD was the only independent predictor of that outcome, with an adjusted odds ratio of 9.3 [8]. Among 5216 hospitalized US veterans, 201 (12%) of those with AKI received KRT, and 47% of patients with AKI had not returned to baseline creatinine by discharge [7].

Post-discharge data are sparser but consistently more favorable. In the Berlin cohort of 74 patients with dialysis-requiring COVID-associated AKI, 36 (49%) died in hospital and one remained hospitalized; among the 37 discharged, the median total duration of KRT was 27 days, and at a median follow-up of 151 days from KRT initiation, 34 (92%) had achieved some degree of kidney recovery, including 23 (62%) with full recovery, while 3 (8%) remained KRT-dependent [23]. In a New York intensive-care cohort of 115 patients requiring RRT, 51% died, 41% recovered kidney function (84% of survivors), and 8% remained on RRT at a median follow-up of 54 days among survivors [35]. In a London intensive-care cohort of 313 critically ill patients, 240 developed AKI and KRT was initiated in 31.9%; dialysis dependence among survivors was 5% at hospital discharge and 4% at 90 days, renal recovery was 81.6% at discharge and 90.9% at 90 days, and 16.5% of AKI survivors met CKD criteria at 90 days, comprising 44.8% of those who had not recovered by discharge against 10.1% of those who had [36]. A subsequent prospective single-center Brazilian cohort of 360 critically ill patients with nephrologist-consulted AKI, 90% of whom required KRT, extended follow-up to one year: mortality was 88.1% at 90 days and 89.3% at one year, and among survivors 72% recovered kidney function, although with a mean eGFR of 65.9 mL/min/1.73 m^2^ against 85.5 mL/min/1.73 m^2^ at baseline [37]. Earlier single-center series point the same way: among Montefiore intensive-care survivors assessed about seven months after discharge, 27 of 35 (77%) patients who had required RRT had been liberated from it and 12 had developed CKD [38], and in a Swedish cohort treated with continuous RRT, no survivor required dialysis at hospital discharge and 73.8% had recovered kidney function at follow-up [39].

Three features of these data should govern their interpretation. First, every figure for dialysis independence is conditioned on survival, and in-hospital mortality in this phenotype ranges from roughly half to two-thirds; a 92% recovery rate among survivors is a 46% recovery rate among all who started KRT. Second, dialysis independence and kidney recovery are not the same endpoint: liberation from KRT is compatible with substantial residual eGFR loss, and the London data show that non-recovery at discharge, even where dialysis was subsequently stopped, predicted CKD at 90 days more than fourfold. Third, follow-up in most of these cohorts does not exceed 90 days, so late transition to maintenance dialysis after initial liberation is largely uncaptured; the one-year Brazilian data indicate that recovery among survivors is common but incomplete [37].

Population-level studies address that gap, although without isolating the dialysis-requiring subgroup. Among 89,216 30-day survivors of COVID-19 in the US Veterans cohort, the adjusted hazard of ESKD was 2.96 (95% CI 2.49–3.51) relative to uninfected controls, graded by acute severity [28]. In England, the hazard of kidney failure was 7.74 (95% CI 7.00–8.56) after hospitalized COVID-19 and remained elevated beyond 180 days [32]. In 3,073,150 propensity-matched pairs in Japan, the hazard of RRT initiation over two years was 2.79 (95% CI 2.56–3.04), with consistent estimates across age, sex, hypertension, diabetes, and COVID-19 severity subgroups [34]. These are population-level hazards, not the probability that a given survivor of dialysis-requiring AKI will progress; however, they establish that the transition to maintenance dialysis after COVID-19 is real, is concentrated after severe disease, and persists well beyond the window in which the cohort studies above stopped counting.

## 6. Reconciling Conflicting Long-Term Evidence

The central disagreement is whether COVID-AKI is intrinsically more nephrotoxic than other-cause AKI. Nugent et al. reported a 14.0 mL/min/1.73 m^2^/year greater adjusted decline after COVID-AKI [24], whereas Aklilu et al. found lower MAKE (adjusted hazard ratio 0.67), driven by lower mortality and less worsened kidney function [29]. These are not mutually exclusive. The earlier cohort required outpatient creatinine testing and measured eGFR slope among survivors; the later used a larger health system cohort, a composite outcome, discharge eGFR as the reference within that outcome, and different comparator eras. Discharge eGFR in the earlier cohort was also higher in the COVID group (68.5 vs. 58.4 mL/min/1.73 m^2^), leaving more room for subsequent decline. Table 3 sets the two against one another feature by feature.

Population-level studies address a different question: whether people with COVID-19 develop more subsequent kidney disease than uninfected individuals or those with other infections. The US Veterans analysis found severity-graded excess kidney outcomes after 30-day survival [28]. A Swedish cohort found a 3.4% adjusted annual eGFR decline after COVID-19 versus 2.3% after other pneumonia, rising to 5.4% after hospitalized COVID-19 [30]. A matched influenza comparison excluding early AKI reported higher 12-month advanced CKD risk [31]. A South Carolina statewide registry of 683,958 people with COVID-19 found a new CKD diagnosis in 1.2%, with intervening AKI being the strongest predictor (hazard ratio 20.7) and no independent association of COVID-19 with CKD once severity of illness and AKI were accounted for [40]. These support post-acute risk but do not prove a COVID-specific mechanism.

The 2025 OpenSAFELY analysis sharpens the message: kidney failure risk was strongly increased after hospitalized COVID-19 and remained elevated beyond 180 days but was not increased after non-hospitalized infection [32]. A 2025 National Clinical Cohort Collaborative study found a bidirectional relationship, with baseline CKD predicting long COVID and long COVID predicting incident CKD and faster eGFR decline [41]. A 2026 Japanese claims study found persistent association with RRT initiation [34]. Three 2025 single-center cohorts extend this evidence beyond the health system datasets that dominate the field. A prospective Congolese cohort of intensive care survivors followed for two years found a steeper adjusted eGFR decline after COVID-associated AKI (−11.09 mL/min/1.73 m^2^/year) and a higher incidence of CKD, reproducing the direction of the Nugent finding in an African setting [42]; a Portuguese cohort of 409 hospitalized patients found AKI to be independently associated with two-year major adverse kidney events (adjusted hazard ratio 1.76) [43], and among 46,448 patients with type 2 diabetes in New York, incident CKD after hospitalized COVID-19 was no more frequent than after hospitalization for other lower respiratory infections, again pointing to illness severity rather than the virus, although in that diabetic population non-hospitalized infection did carry excess risk relative to uninfected controls, a finding not seen in the OpenSAFELY analysis [44]. A companion analysis in type 1 diabetes found a higher incidence of new CKD after both hospitalized and non-hospitalized COVID-19 relative to uninfected controls over up to 40 months [45]. Risk is real but concentrated; universal screening after mild infection is not supported.

## 7. Predictors of Non-Recovery

Predictors of incomplete recovery fall into six domains differing markedly in how well established they are and what they imply for practice. Table 4 gives each with the strength of evidence and clinical implication, separating findings reproduced across cohorts from those resting on single studies awaiting validation. A 36-month analysis of 1747 patients hospitalized during 2020 illustrates the point: AKI together with a hospital stay of 27 days or more identified those at highest risk of rapid post-discharge eGFR decline, and the pattern held irrespective of COVID-19 status [46].

## 8. Biomarkers: Promise Without Clinical Readiness

Serum creatinine and urine output remain the clinical standard despite delayed kinetics and sensitivity to muscle mass, volume status, and measurement frequency. Cystatin C was associated with critical illness and mortality in a 481-patient cohort but is also influenced by inflammation and corticosteroids [47]. Small emergency-department studies found similar AKI discrimination for creatinine and cystatin C, with acceptable performance for neutrophil-gelatinase-associated lipocalin (NGAL) [48]. None establishes a COVID-specific threshold or incremental benefit over routine clinical data.

Three classes of biomarker must be distinguished, because the evidence base differs for each and the distinction is routinely blurred. In the FDA–NIH Biomarkers, EndpointS, and other Tools (BEST) taxonomy, a diagnostic biomarker detects the presence of a condition, here AKI or tubular injury, and is judged by discrimination against a reference standard. A prognostic biomarker estimates the likelihood of a subsequent clinical event in patients who already have the condition, irrespective of any treatment; prediction of death, need for dialysis, persistent AKI, or eventual kidney recovery all belong to this class. A predictive biomarker identifies patients who will benefit from a specific intervention and can be validated only within a treatment comparison. Nearly all evidence for COVID-associated AKI concerns the first two classes and, within the second, concerns short-horizon events: incident AKI, requirement for KRT, and in-hospital death. Association with those outcomes is not evidence that a marker forecasts long-term kidney recovery, which is a separate prognostic question with a different reference standard and a follow-up window measured in months. Only one small study addresses it directly [49], and a second addresses persistent AKI at about one week rather than long-term recovery [50]; no biomarker has been evaluated as predictive in this population, because no intervention trial has stratified by biomarker status.

Admission soluble urokinase plasminogen activator receptor (suPAR) was strongly associated with incident AKI and dialysis in a multinational cohort, and an ICU study linked suPAR with severe AKI and mortality [51,52]. Later work evaluated combinations of suPAR, soluble TNF receptors, endostatin, s-RAGE, and host-response proteins [50,53,54,55]. Urinary NGAL shows modest discriminative value for AKI, whereas kidney injury molecule-1 (KIM-1), vanin-1, calprotectin, and tubular stress panels are inconsistent [56,57]; all of these are diagnostic or short-horizon prognostic findings. A comparator study found overlapping severe AKI signatures in COVID and non-COVID respiratory illness, reinforcing nonspecificity [15]. None of the associations in this paragraph was measured against kidney recovery.

The hierarchy is therefore unambiguous. Conventional assessment by serum creatinine, urine output, AKI stage, and baseline kidney function remains the standard against which every candidate must be judged, and none has yet shown incremental value over it. To change practice, a biomarker should improve discrimination, calibration, or decision-making beyond AKI stage, baseline eGFR, urine output, and illness severity; use a reproducible assay and threshold; be externally validated; and show clinical utility. Current evidence does not meet that standard, and small studies of recovery markers such as urinary SerpinA3 are hypothesis-generating [49]. Table 5 summarizes the candidates against these criteria.

## 9. Special Populations and Vaccination Context

Advanced CKD, maintenance dialysis, and kidney transplantation identify patients with reduced kidney reserve and disproportionate severe COVID risk. Early European registry data showed approximately 20% 28-day mortality in dialysis and transplant recipients, with adjusted comparisons suggesting higher risk after transplantation [58]. A transplant meta-analysis reported substantial AKI and mortality but reflected early, unvaccinated cohorts [59]. Mortality later declined across waves, less so where vaccine responses were impaired [60], and updated vaccination remained protective against hospitalization and death among US dialysis patients, with waning [61].

Saudi evidence adds regional relevance but is dominated by acute, early-wave outcomes. A five-center cohort of 130 kidney transplant recipients reported AKI in 26.9%, mortality in 10.8%, and graft loss in 0.77% [62]. A smaller southern Saudi cohort included only 31 infected recipients, of whom four of five ICU patients required dialysis and died; it should be read with exact denominators and not generalized [63]. Saudi multicenter data on post-discharge eGFR trajectories and vaccine-era recovery remain a clear gap. Longer-term transplant data are now emerging elsewhere: in a Brazilian cohort of 43 recipients hospitalized with COVID-19 and followed for a median of 2.5 years, AKI during admission predicted a steeper subsequent decline in graft function (−6.99 mL/min/1.73 m^2^/year) [64]. By contrast, among 148 Chinese recipients infected predominantly with the Omicron variant and followed for nine months, most with mild disease, GFR was essentially unchanged [65], consistent with graft injury tracking illness severity rather than infection itself. In the Omicron era, a prospective Chinese cohort of 187 discharged patients with diabetes and CKD reported 35.8% rapid CKD progression and 26.7% mortality within one year, with baseline eGFR below 60 mL/min/1.73 m^2^ being the independent predictor of progression [66].

Vaccination matters here only as far as it modifies the risk gradient on which surveillance priority depends. A large direct comparison found substantially more AKI after infection than after vaccination; nationwide data showed no increase in new-onset glomerulonephritis, although a small short-term relapse signal has been reported in established glomerular disease [67,68,69]. CureGN data associated infection, but not vaccination, with worsening disease activity, and post-vaccination gross hematuria in IgA nephropathy generally did not produce a significant two-year eGFR difference [70,71]. These findings support vaccination while justifying prompt evaluation of new hematuria, edema, oliguria, or systemic symptoms. Vaccination status belongs in risk assessment because it alters the probability of severe, hospitalized disease, not because vaccination itself warrants kidney monitoring.

## 10. Proposed Post-COVID Renal Surveillance

No trial has validated a surveillance schedule specific to COVID-19-associated AKI. The framework proposed here is therefore not a new recommendation but an application of existing post-AKI guidance to a population whose severity gradient is now well characterized. The Kidney Disease: Improving Global Outcomes (KDIGO) guideline already advises evaluation three months after AKI for resolution, new-onset, or worsening CKD, and that those without CKD then be regarded as at increased risk [72]. The COVID-19 literature adds a basis for prioritization within that schedule: the gradient across non-hospitalized infection, hospitalization, and critical illness identifies who most warrants that assessment. Highest priority goes to stage 2–3 AKI, KRT, incomplete creatinine recovery, substantial albuminuria or active sediment, advanced CKD, dialysis, or transplantation.

**Figure 2:**
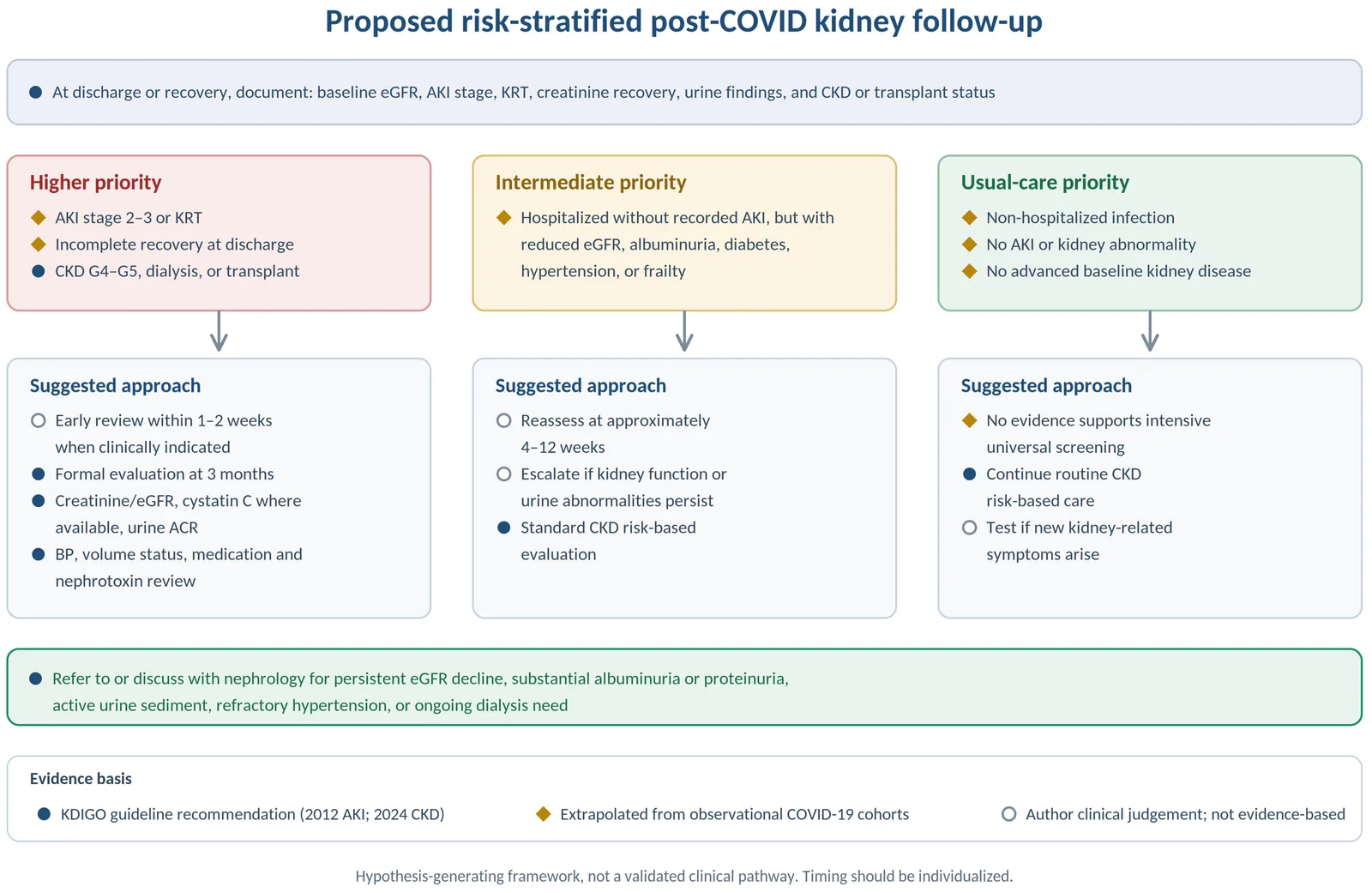
Proposed risk-stratified framework for post-COVID kidney follow-up, with each element marked by its evidence basis. Filled circles denote KDIGO guideline recommendations (2012 AKI; 2024 CKD) [72,73]; diamonds denote elements extrapolated from observational COVID-19 cohorts; open circles denote author clinical judgment without direct evidentiary support. The framework is hypothesis-generating and has not been prospectively validated.

A practical review includes serum creatinine and eGFR, urine albumin-to-creatinine ratio, blood pressure, volume status, medication reconciliation, avoidance of unnecessary nephrotoxins, and assessment of dialysis dependence. Nephrology input suits persistent decline, substantial proteinuria, active sediment, refractory hypertension, uncertain baseline function, or ongoing KRT. Hospitalized patients without recorded AKI but with reduced baseline eGFR, albuminuria, diabetes, hypertension, or frailty merit risk-based reassessment; evidence does not support universal screening after uncomplicated non-hospitalized infection. These correspond to standard post-AKI and CKD evaluation rather than COVID-specific testing [72,73]. Where available, KDIGO now recommends estimating GFR from creatinine and cystatin C combined, directly relevant to critical illness survivors in whom muscle loss makes creatinine unreliable [73]. Figure 2 presents the framework with each element marked by its evidence basis, because the elements are not equally supported and should not be read as if they were. Three tiers are distinguished. The first comprises KDIGO guideline recommendations that apply to any AKI survivor regardless of cause: documentation of baseline function and AKI characteristics, evaluation at three months, assessment by eGFR and urine albumin-to-creatinine ratio with combined creatinine–cystatin C estimation where available, blood pressure and nephrotoxin review, standard CKD risk-based care, and the criteria for nephrology referral [72,73]. The second comprises elements extrapolated from observational COVID-19 cohorts rather than from guideline or trial evidence: the stratification itself, which rests on the severity gradient documented in Section 5 and Section 6, and the conclusion that intensive universal screening after non-hospitalized infection is unsupported, which rests principally on the OpenSAFELY analysis [32]. The third comprises author clinical judgment with no direct evidentiary basis: the suggestion of an early review within one to two weeks of discharge for the highest-priority group, the four-to-twelve-week reassessment window for the intermediate group, and the threshold at which to escalate. These time points are offered as reasonable starting positions for prospective evaluation, not as recommendations. The framework as a whole is therefore hypothesis-generating: its guideline-derived components apply irrespective of COVID-19, its COVID-derived components identify who most warrants those existing recommendations, and the schedule that combines them has not been tested.

The first major revision of the KDIGO AKI guidance, the KDIGO 2026 Clinical Practice Guideline for Acute Kidney Injury and Acute Kidney Disease, completed public review in May 2026 and is being prepared for publication [74]. It treats AKI and AKD as a continuum of injury, recovery, and CKD risk, and incorporates structural biomarkers alongside functional criteria. Post-COVID surveillance proposals should be reconciled with the final version once published.

## 11. Therapeutic Implications of Persistent Post-COVID Kidney Dysfunction

Surveillance is justifiable only if detection changes management, a point usually left implicit in the post-COVID kidney literature, which characterizes risk in detail and stops short of therapy. Not every reduction in eGFR after AKI constitutes CKD. KDIGO defines CKD as abnormalities of kidney structure or function present for a minimum of three months with implications for health, requiring either a GFR below 60 mL/min/1.73 m^2^ or a marker of kidney damage such as an albumin-to-creatinine ratio of at least 30 mg/g, urine sediment abnormalities, electrolyte disturbance from tubular disorders, or a structural or histological abnormality [73]. KDIGO further advises that, in patients recovering from AKI, the diagnosis of CKD should be deferred until at least three months after the AKI episode, once kidney function has had the opportunity to stabilize [73]. A survivor meeting those criteria at that point has CKD and becomes eligible for guideline-directed therapy on the same basis as anyone else; a survivor with a modest, stable eGFR reduction that does not cross those thresholds does not.

That therapy is well defined, but one point must be stated before any of it is listed: every element below is supported by trials in general CKD populations and none by a trial enrolling patients on the basis of recent COVID-associated AKI. What follows is therefore what current guidance recommends for CKD, not what has been shown to work after COVID-19. Renin–angiotensin system (RAS) inhibition remains first-line for blood pressure control and albuminuria reduction. Sodium–glucose cotransporter-2 inhibition is recommended for adults with type 2 diabetes and CKD at eGFR above 20 mL/min/1.73 m^2^ and independently of diabetes at the same threshold with a urine albumin-to-creatinine ratio above 200 mg/g or heart failure at any albuminuria [73]. A non-steroidal mineralocorticoid receptor antagonist is suggested for type 2 diabetes, eGFR above 25 mL/min/1.73 m^2^, normal potassium, and albuminuria persisting despite a maximally tolerated RAS inhibitor [73,75]. Nephrotoxin stewardship applies throughout.

The extrapolation deserves more than acknowledgment because its implications are concrete. The pivotal SGLT2 inhibitor and finerenone trials enrolled patients with established, stable CKD, typically years from diagnosis and selected for stable eGFR at entry; post-AKI survivors have recovering and often unstable function, an uncertain baseline, and a competing risk of death that the trial populations did not carry. Effect sizes measured in the former may not transfer to the latter, the benefit–risk balance during the recovery phase is unknown, and the optimal timing of initiation has never been tested. None of this argues against treatment; it argues against assuming that the evidence is stronger than it is. Two further caveats follow. First, applying CKD trial evidence to this population remains extrapolation, defensible but untested. Second, both RAS and SGLT2 inhibition produce an expected early eGFR decline that is hard to distinguish from ongoing post-AKI deterioration when baseline is uncertain. This argues for starting therapy once the trajectory has stabilized rather than at discharge and for documenting a credible reference eGFR first.

The resulting research question is more specific than whether COVID-19 causes CKD. It is whether structured post-AKI follow-up that leads to earlier initiation of guideline-directed CKD therapy improves kidney outcomes in survivors of severe COVID-19, relative to usual care. That is a trial design, and it is the logical successor to the observational literature reviewed here. The evidence gaps and research priorities are summarized in Table 6.

## 12. Limitations of the Review

This synthesis has several constraints. Its database search was deliberately narrow, combining COVID-19 with a single phrase in one bibliographic database, and contributed only two of the included primary studies; a structured query run at revision retrieved the principal cohorts and, after abstract screening of the 75 records whose titles indicated longitudinal or kidney function outcomes, added thirteen studies, but the remaining 235 records were classified by title index terms only and not individually screened; the evidence base was assembled predominantly by hand-searching reference lists and by author selection, so relevant reports not cited by the sources consulted or not known to the authors may have been missed, and only English-language publications were used. Selection and interpretation were performed by one author without duplicate review, and no formal risk-of-bias instrument was applied; the design appraisal in Table 2 applies four prespecified features consistently across cohorts but is not a validated risk-of-bias score. No quantitative pooling was attempted, and the reconciliation in Section 6 is an argument about study design rather than a statistical result. The literature is dominated by North American and European health system cohorts, although single-center cohorts from Africa, South America, and Asia are now appearing [37,42,66], and the framework in Section 10 has not been prospectively evaluated. Sex-disaggregated outcomes were reported inconsistently, and most cohorts were underpowered for sex differences, so sex-stratified analysis was not possible and remains a research priority. Finally, early-pandemic estimates should not be treated as current incidence figures as vaccine-era and Omicron-era cohorts mature.

## 13. Conclusions

COVID-19-associated AKI marks a group at increased risk, but long-term trajectories are heterogeneous and cannot be attributed to SARS-CoV-2 alone. AKI severity, duration, KRT, lower baseline eGFR, and incomplete early recovery are the most reproducible risk indicators. Comparative studies disagree on whether COVID-AKI is more harmful than other-cause AKI because they measure different populations, baselines, and outcomes; population data nevertheless concentrate risk after hospitalization and severe disease. Biomarkers remain investigational. Until intervention studies exist, care should prioritize hospitalized AKI, incomplete recovery, advanced CKD, dialysis, and transplantation rather than universal surveillance.

## Figures and Tables

**Table 2:** Longitudinal cohorts and syntheses of kidney outcomes after COVID-19 or COVID-19-associated AKI, with four design features (kidney reference point, analytic population, outcome ascertainment, and handling of competing death) applied to each.

Study	Population	Follow-Up/Outcome	Main Finding	Design Appraisal (Reference Point; Population; Ascertainment; Competing Death)
Nugent et al., 2021 [24]	182 COVID-AKI; 1430 non-COVID AKI	Post-discharge eGFR slope	Steeper adjusted eGFR decline after COVID-AKI	Pre-illness outpatient baseline; survivors with follow-up creatinine; laboratory; death not modeled
Bowe et al., 2021 [28]	89,216 survivors; 1.64 million controls	Post-acute kidney outcomes	Severity-graded excess AKI, eGFR loss, ESKD, MAKE	Recorded baseline eGFR; 30-day survivors vs. uninfected; EHR and laboratory; death within MAKE, inverse-probability weighting
Tan et al., 2023 [25]	12,891 hospitalized patients	Up to 1 year	Severe AKI impairs recovery at 180 and 365 days	Pre-admission creatinine within 365 days required; hospitalized with baseline; EHR laboratory; mortality modeled separately
Lu et al., 2023 [22]	3296 survivors with hospital AKI	90 days, 1 year	Longer recovery predicts MAKE and CKD	Hospital baseline; AKI survivors stratified by recovery time; EHR; death within MAKE
Aklilu et al., 2024 [29]	987 COVID-AKI within 9624	Up to 2 years	Lower adjusted MAKE after COVID-AKI	Discharge eGFR; survivors to discharge; EHR; death within MAKE composite
Mahalingasivam et al., 2024 [30]	134,565 COVID; 35,987 pneumonia	Longitudinal eGFR	Steeper annual decline, especially if hospitalized	Pre-infection routine eGFR; infected vs. pneumonia, not survivor-restricted; laboratory; —
Chen et al., 2025 [31]	141,587 matched pairs, no early AKI	12 months	More advanced CKD than influenza	—; matched, early AKI excluded; coded diagnoses; —
OpenSAFELY, 2025 [32]	National matched cohort, England	>180 days	Kidney failure raised after hospitalization only	Primary-care eGFR; matched general population; coded EHR; cause-specific Cox, competing risk handled
Frediani et al., 2025 [3]	Scoping review: 37 studies; 1.31 million	Variable	AKI linked to renal long COVID	Not applicable (evidence synthesis)
Ribeiro et al., 2025 [33]	655 hospitalized survivors	Mean 6.5 months	Late dysfunction in 28%, strongest after community AKI	—; hospitalized survivors, prospective; clinical follow-up; survivors only
Miyamori et al., 2026 [34]	3,073,150 matched pairs	2 years	Higher RRT initiation	No kidney reference (claims); matched general population; claims for RRT initiation; inverse-probability censoring weighting

Note: Sources are cited in column 1. The appraisal column records, in order, the kidney reference point, the analytic population and its survivor conditioning, the mode of outcome ascertainment, and the handling of competing death; — denotes a feature not reported in the primary publication. Abbreviations: EHR, electronic health record; ESKD, end-stage kidney disease; MAKE, major adverse kidney events; RRT, renal replacement therapy.

**Table 3:** Design features accounting for divergent findings between the two principal comparative studies of COVID-associated versus other-cause AKI.

Design Feature	Nugent et al., 2021 [24]	Aklilu et al., 2024 [29]
Setting	Five hospitals, one health system	Larger multi-hospital health-system cohort
Sample	182 vs. 1430 non-COVID AKI	987 within 9624 AKI
Enrollment era	First wave, pre-vaccination	Later, partly vaccinated era
Kidney reference point	Pre-illness baseline defines AKI; slope measured from discharge	Discharge eGFR
Primary outcome	eGFR slope after discharge	Composite MAKE
Ascertainment requirement	Outpatient creatinine testing required	Health system records
Analytic population	Survivors with follow-up testing	Broader survivor cohort
Competing death	Not incorporated	Mortality within composite
Direction of finding	Steeper decline after COVID-AKI	Lower MAKE after COVID-AKI

Note: Reading the table by row rather than column shows that the two studies are not measuring the same quantity. A slope estimated only among patients returning for outpatient testing is conditioned on survival and engagement with care; a composite including death improves whenever mortality falls, as it did across waves. Using discharge rather than pre-illness eGFR as reference systematically reduces the apparent loss, because discharge values already incorporate the injury. None of these is a flaw, yet together they suffice to reverse the apparent direction of effect without either result being wrong. The same four design features explain most of the remaining heterogeneity across the cohorts, and the appraisal column of Table 2 applies them to every study rather than to the two comparative ones alone. On the kidney reference point, only Nugent et al. [24] and Tan et al. [25] anchor to a pre-illness measurement, and Tan et al. required one for inclusion; Aklilu et al. [29] use discharge eGFR, which already incorporates the acute injury and so understates subsequent loss; the population-level studies use routine pre-infection values or, in the claims-based analysis, no kidney reference at all. On analytic population, the hospital cohorts are survivor-conditioned to differing degrees, from survival to discharge to survival with a follow-up creatinine, whereas the population-level studies compare infected individuals with uninfected or pneumonia comparators and are not survivor-restricted. On ascertainment, laboratory eGFR trajectories, coded diagnoses, and claims for RRT initiation measure different constructs and cannot be pooled. On competing death, the composite MAKE outcomes fold mortality into the estimate, the cause-specific and inverse-probability-weighted models handle it explicitly, and the slope-based analyses do not model it. Read across the rows, the pattern is the one already identified for the two comparative studies: the designs most favorable to a COVID-specific excess combine survivor-conditioned populations, laboratory outcomes, and no competing-risk adjustment, and the designs least favorable use composite outcomes that include death. That is a statement about design rather than biology, and this review declines to pool.

**Table 4:** Factors associated with incomplete recovery or adverse post-acute kidney trajectories.

Domain	Examples	Evidence Interpretation	Clinical Implication
AKI severity and KRT	Stage 3 AKI, dialysis, oliguria	Consistent across cohorts	Highest priority for early reassessment
Duration/discharge recovery	Prolonged AKI; creatinine above baseline at discharge	Predicts MAKE, recurrent AKI, CKD	Use a credible pre-illness baseline; document recovery
Baseline kidney reserve	Lower eGFR, albuminuria, CKD	Linked to AKI, non-recovery, severe COVID	Do not attribute progression solely to SARS-CoV-2
Acute illness severity	ICU, ARDS, ventilation, instability	May mediate AKI and later decline	Compare with non-COVID critical illness
Host factors	Age, diabetes, hypertension, cardiovascular disease	Recurrent across cohorts	Manage as standard CKD risk
AKI phenotype	Community- vs. hospital-acquired AKI	One cohort: stronger late risk if community-acquired	Needs external validation
Social and structural context	Access, follow-up intensity, deprivation, ethnicity	Affect exposure, severity, ascertainment	Race is not a mechanism or an APOL1 proxy

AKI, acute kidney injury; APOL1, apolipoprotein L1; ARDS, acute respiratory distress syndrome; CKD, chronic kidney disease; eGFR, estimated glomerular filtration rate; MAKE, major adverse kidney events.

**Table 5:** Candidate biomarkers for COVID-19-associated AKI, classified by biomarker class and by whether any evidence bears on long-term kidney recovery. BEST, Biomarkers, EndpointS, and other Tools (FDA–NIH) taxonomy *.

Marker	Biomarker Class (BEST)	What Has Been Shown	Evidence for Long-Term Recovery	Readiness
Creatinine/urine output	Diagnostic; prognostic when trended	AKI detection and staging; recovery trajectory defines phenotype	Time to creatinine recovery predicts 90-day MAKE and new CKD [22]	Clinical standard
Cystatin C	Diagnostic (GFR estimation)	Associated with critical illness and mortality [47]; AKI discrimination similar to creatinine [48]; confounded by inflammation and corticosteroids	None	Adjunct to eGFR per KDIGO 2024; not COVID-specific
suPAR	Prognostic (AKI, KRT, death)	Strong association with incident AKI and dialysis [51] and with severe AKI and mortality [52]	None	Investigational
NGAL (serum or urine)	Diagnostic (tubular injury)	Modest AKI discrimination; specimen and threshold vary [48,56,57]	None	Investigational
KIM-1, vanin-1, calprotectin	Diagnostic or prognostic (tubular, inflammatory)	Inconsistent; calprotectin moderately predictive of ICU admission only [56]	None	Investigational
sTNFR1/2, endostatin, s-RAGE	Prognostic (AKI, dialysis, death)	Single-center associations [53,54,55]; little external validation	None	Investigational
Host-response protein panels	Prognostic (persistent AKI)	Signals for AKI persisting at about one week in critically ill COVID-19 [50]; small samples	Persistent AKI, not long-term recovery	Research use only
Urinary SerpinA3	Prognostic (recovery); candidate	Single small prospective study [49]	Only marker evaluated against recovery; hypothesis-generating	Research use only
Any marker	Predictive (treatment benefit)	No study has tested whether any biomarker identifies who benefits from an intervention	None	Not evaluated

* Three classes of biomarker must be distinguished, because the evidence base differs for each and the distinction is routinely blurred. In the FDA–NIH Biomarkers, EndpointS, and other Tools (BEST) taxonomy, a diagnostic biomarker detects the presence of a condition, here AKI or tubular injury, and is judged by discrimination against a reference standard. A prognostic biomarker estimates the likelihood of a subsequent clinical event in patients who already have the condition, irrespective of any treatment; prediction of death, need for dialysis, persistent AKI, or eventual kidney recovery all belong to this class. A predictive biomarker identifies patients who will benefit from a specific intervention and can be validated only within a treatment comparison. Nearly all evidence for COVID-associated AKI concerns the first two classes and, within the second, concerns short-horizon events: incident AKI, requirement for KRT, and in-hospital death. Association with those outcomes is not evidence that a marker forecasts long-term kidney recovery, which is a separate prognostic question with a different reference standard and a follow-up window measured in months. Only one small study addresses it directly [49], and a second addresses persistent AKI at about one week rather than long-term recovery [50]; no biomarker has been evaluated as predictive in this population, because no intervention trial has stratified by biomarker status.

**Table 6:** Summary of the evidence gaps and research priorities.

Adopt standardized AKI, AKD, recovery, and MAKE definitions with a credible pre-infection baseline and competing risk methods. Use contemporaneous non-COVID comparators to test COVID specificity. Extend follow-up beyond 12 months, including eGFR slope, albuminuria, recurrent AKI, dialysis dependence, and cardiovascular events. Validate risk models and biomarkers externally, and test whether biomarker-guided care improves outcomes. Evaluate surveillance strategies, not only associations: timing, referral thresholds, uptake, cost-effectiveness. Build multicenter vaccine- and Omicron-era cohorts in underrepresented regions.

## Data Availability

All evidence discussed is available in the cited publications.

## Supplementary Materials

The following supporting information can be downloaded at: https://www.mdpi.com/article/10.3390/pathogens15101022/s1. Figure S1: study flow diagram.
